# Transient receptor potential vanilloid 4 (TRPV4) silencing in *Helicobacter pylori‐*infected human gastric epithelium

**DOI:** 10.1111/hel.12361

**Published:** 2016-09-30

**Authors:** Hiroshi Mihara, Nobuhiro Suzuki, Jibran Sualeh Muhammad, Sohachi Nanjo, Takayuki Ando, Haruka Fujinami, Shinya Kajiura, Ayumu Hosokawa, Toshiro Sugiyama

**Affiliations:** ^1^ Department of GastroenterologyGraduate School of Medicine and Pharmaceutical SciencesUniversity of ToyamaToyamaJapan; ^2^ Center for Medical Education and Career DevelopmentUniversity of ToyamaToyamaJapan; ^3^ Department of Biological and Biomedical SciencesFaculty of Health SciencesThe Aga Khan UniversityKarachiPakistan

**Keywords:** gastric epithelium, *Helicobacter pylori*, methylation silencing, transient receptor potential vanilloid 4, TRPV4

## Abstract

**Background:**

*Helicobacter pylori* (HP) infection induces methylation silencing of specific genes in gastric epithelium. Various stimuli activate the nonselective cation channel TRPV4, which is expressed in gastric epithelium where it detects mechanical stimuli and promotes ATP release. As CpG islands in *TRPV4* are methylated in HP‐infected gastric epithelium, we evaluated HP infection‐dependent changes in *TRPV4* expression in gastric epithelium.

**Materials and Methods:**

Human gastric biopsy samples, a human gastric cancer cell line (AGS), and a normal gastric epithelial cell line (GES‐1) were used to detect *TRPV4* mRNA and protein expression by RT‐PCR and Western blotting, respectively. Ca^2+^ imaging was used to evaluate TRPV4 ion channel activity. *TRPV4* methylation status was assessed by methylation‐specific PCR (MSP). ATP release was measured by a luciferin‐luciferase assay.

**Results:**

TRPV4 mRNA and protein were detected in human gastric biopsy samples and in GES‐1 cells. MSP and demethylation assays showed *TRPV4* methylation silencing in AGS cells. HP coculture directly induced methylation silencing of *TRPV4* in GES‐1 cells. In human samples, HP infection was associated with *TRPV4* methylation silencing that recovered after HP eradication in a time‐dependent manner.

**Conclusion:**

HP infection‐dependent DNA methylation suppressed *TRPV4* expression in human gastric epithelia, suggesting that *TRPV4* methylation may be involved in HP‐associated dyspepsia.

## 1 Introduction

*Helicobacter pylori* (HP) is highly adapted to the gastric environment and infects approximately 50% of the world's human population.^1^ HP damages the underlying gastric mucosa and initiates a chronic inflammatory reaction by adhering to the gastric epithelium, which further extends gastric tissue injury. There are several reports that associated HP infection with events that cause chronic gastritis, intestinal metaplasia, gastric adenocarcinoma, and dyspepsia.^2^, ^3^

DNA methylation is an epigenetic modification that in mammals occurs at cytosine residues, and predominantly in the context of CpG dinucleotides. CpG islands are regions where CpG methylation within gene promoters can lead to their silencing. In contrast, CpG hypomethylation has been associated with gene overexpression. Recent studies suggested that *H. pylori* (HP) infection induces methylation silencing^4^, ^5^ and that HP eradication would decrease DNA methylation in a gene‐specific manner.^6^

The transient receptor potential vanilloid 4 channel (TRPV4) is a nonselective cation channel that is involved in various cellular functions^7^ and is activated by several physical stimuli, such as heat and mechanical, as well as endogenous or exogenous stimuli (eg, endogenous arachidonic acid metabolites, 5,6‐EET) 8 and the specific agonist GSK1016790A.^9^ TRPV4 is widely expressed throughout the gastrointestinal epithelium, including the esophagus, intestine, and gastric epithelium where it contributes to adenosine triphosphate (ATP) release via exocytosis.^10^, ^11^ We recently reported that TRPV4 is expressed in mouse and rat gastric epithelium and contributes to ATP release and gastric emptying.^12^ However, to the best of our knowledge, to date, there are no reports on TRPV4 expression analysis in human gastric epithelium.

Recent studies revealed that TRPV4 function is affected by gain‐ or loss‐of‐function mutations, membrane trafficking, or gain of channel function itself.^7^, ^13^, ^14^, ^15^, ^16^ In addition, TRPV4 expression is downregulated in some cancers by an unknown mechanism.^17^, ^18^ However, epigenetic modulations of TRPV4 expression have not been reported. We thus hypothesized that gastric *TRPV4* expression is suppressed by DNA methylation associated with HP infection.

## 2 Methods

### 2.1 Cell lines

The AGS cancer cell line (CCL‐248; American Type Culture Collection, Manassas, VA, USA) was cultured in Dulbecco's modified Eagle medium supplemented with 10% heat‐inactivated fetal bovine serum, 100 μg/mL streptomycin, and 100 U/mL penicillin. AGS cells were maintained in a humidified incubator at 37°C. The GES‐1 gastric epithelial cell line was obtained from The University of Texas at Austin. GES‐1 cells are derived from a human nontumorigenic gastric mucosa epithelium and immortalized via SV40.^19^ GES‐1 cells were maintained in RPMI supplemented with 10% fetal bovine serum, 1% glutamate, and 1% penicillin‐streptomycin.

### 2.2 Bacterial strains and culture, coculture conditions

The HP strain 193C originating from a patient with gastric cancer^20^ and the HP strain NCTC developed from an ATCC strain at Yamaguchi University Hospital, Japan,^21^ were used in this study. HP were cultured in Brucella broth medium (BB) supplemented with 10% fetal bovine serum (FBS) under microaerophilic conditions (5% O_2_, 10% CO_2_, and 85% N_2_ at 37°C; Sanyo‐Multigas Incubator; SANYO Electric Co., Ltd. Tokyo, Japan) with 100% humidity on a gyratory shaker (Thermo‐shaker; Thermonics, Tokyo, Japan) at 160 reciprocations per min. The formula wherein absorbance of 0.1=10^8^ bacteria/mL was used to estimate the concentration of bacteria in each culture.

GES‐1 and HP coculture was carried out as described previously.^22^ Briefly, GES‐1 cells were seeded onto 10‐cm culture dishes and grown for 24 hours; these cultures were then washed with phosphate‐buffered saline (PBS) three times before coculture. Fresh RPMI 1640 medium without antibiotics or FBS was added 1 hour before addition of HP. HP was cultured overnight in BB‐FBS 10% under the conditions described above and then washed twice with PBS. Bacteria were then directly added to the gastric cells at a bacterium/cell ratio of 50:1 for the indicated times.

### 2.3 Human gastric biopsy samples

We obtained gastric biopsy samples from healthy individuals with/without *H. pylori* infection or patients in whom HP was successfully eradicated through esophagogastroduodenoscopy at Toyama University Hospital in Japan. HP infection was defined as more than one positive result for antisera, urease assay, or microscopic evaluation, and no infection was defined as at least two negative results and no obvious atrophic gastritis, which was judged by two endoscopists certified by the Japanese Gastroenterological Endoscopy Society. Tissue sample analysis procedures were approved by the University of Toyama human subjects committee, and written informed consent was obtained from all individuals. We enrolled individuals between ages 37 and 77 to minimize background factors among the three groups (HP‐negative, HP‐positive, and HP‐eradicated groups) (Table 1).

**Table 1:** Study subject demographic characteristics

	HP−	HP+	Erad	*P* value
n	9	10	12	
Sex (m;%)	77.8	70.0	75.0	*NS*
Age (median)	47‐69 (56)	57‐77 (63)	37‐71 (65.6)	NS

### 2.4 Reverse transcription PCR analysis

RT‐PCR was performed as previously described.^10^, ^23^ Total RNA (1 μg) was isolated using the RNeasy Mini Kit (Qiagen, Courtaboeuf, France). PCR was performed using rTaq DNA polymerase (TaKaRa) or FX neo (TOYOBO) in an iCycler (Bio‐Rad Laboratories, CA, USA) with specific primer sets (Table S1). PCR conditions used for FX neo were one cycle at 94°C for 2 minutes, 40 cycles at 98°C for 10 seconds, 55°C for 30 seconds, and 68°C for 90 seconds, followed by one cycle at 72°C for 2 minutes. PCR products for human TRPV4 in human gastric biopsy samples and GES‐1 were sequenced using an ABI3500 sequencer (Applied Biosystems, Foster City, CA, USA). A BLAST search (https://blast.ncbi.hlm.nih.gov/Blast.cgi?PAGE_TYPE=BlastSearch) confirmed that the sequences were consistent with reported TRPV4 cDNA sequences. Quantitative RT‐PCR was performed using a LightCycler 480 SYBR Green I Master apparatus (Roche, Meylan, France). Cycling conditions were 94°C for 5 minutes followed by 40 cycles of 94°C for 15 seconds and 60°C for 30 seconds. Data were collected and analyzed by quantification relative to beta‐actin.

### 2.5 Immunochemistry and Western blotting

Immunochemistry and Western blotting were performed as previously described 10 using the antibodies summarized in Table S2. Human gastric (corpus) biopsies were fixed at 4°C for 6 hours. Tissues were placed in PBS–sucrose and embedded in OCT compound (Tissue Tek, Elkhart, IN, USA). Nonspecific antibody binding was reduced by incubation in BlockAce (Yukijirushi, Sapporo, Japan) for 1 hour at room temperature prior to antibody exposure. Antigenic peptides for absorption experiments were purchased from Abcam. Preparations were analyzed using a confocal laser scanning microscope (LSM 700, Carl Zeiss Thornwood, New York). For Western blotting, AGS or GES‐1 cell lysates were resolved by SDS‐PAGE on 7.5% SDS–polyacrylamide gels and transferred to polyvinylidene membranes. The membrane was blocked in BlockAce and probed with primary antibodies (Table S2). Immunopositive bands were visualized with the ECL system (Thermo Fisher Scientific, MA, USA).

### 2.6 Ca^2+^ imaging

Fura‐2 fluorescence was measured in AGS cells with a standard bath solution containing 140 mM NaCl, 5 mM KCl, 2 mM MgCl_2_, 2 mM CaCl_2_, 10 mM HEPES, and 10 mM glucose at pH 7.4 (adjusted with NaOH) at 25°C. Results are presented as ratios of fluorescence intensities obtained with fura‐2 emissions at 340 nm and 380 nm. GSK1016790A^9^ (Sigma–Aldrich Corporation, MO, USA) was used as a TRPV4 agonist. *F*_340_/*F*_380_ was calculated and acquired with an image processing system (AQUA COSMOS, Hamamatsu Co., Hamamatsh City, Japan). Changes in ratio (Δ) were calculated by subtracting the mean basal values from peak values.

### 2.7 Demethylation assay and methylation‐specific PCR (MSP)

Demethylation assays were performed using two kinds of demethylating agents, 5‐azacytidine (5‐Aza, 10 μM) or 5‐aza‐2′‐deoxycitidine (5‐AzaDC) (1 μM) with a 1‐week incubation. Human gastric biopsies were placed in RNAlater (Invitrogen) and stored at −80°C. Genomic DNA was isolated using a previously described method.^4^ Fully unmethylated DNA was prepared by amplifying genomic DNA with the GenomiPhi amplification system (GE Healthcare, Buckinghamshire, UK). Twenty‐one primer pairs near the *TRPV4* transcription start site were designed either manually or with Methyl Primer Express v1.0 provided by Applied Biosystems. The most relevant primer pairs are listed in Supporting Table 1. Methylation‐specific PCR (MSP) was performed using the primer sets with AmpliTaq Gold (Applied Biosystems) in an iCycler device (Bio‐Rad). PCR conditions used were one cycle at 94°C for 10 minutes, 33 cycles at 94°C for 30 seconds, 56.7°C for 30 second, and 72°C for 30 seconds followed by one cycle at 72°C for 2 minutes.

### 2.8 ATP release measurement

ATP concentrations released from AGS cells cultured in 12‐well plates were measured by a luciferin‐luciferase assay (ATP Bioluminescence assay kit CLS II, Roche Diagnostics) and a luminometer (Lumat LB 9507, Berthold Technologies, Japan), using a previously described method.^12^ For chemical stimuli, cells cultured to 70%‐80% confluence and incubated in 500 μL bath solution for 30 minutes at room temperature (25°C) were used to measure basal ATP release. The supernatant was collected and replaced gently with another 500 μL of bath solution with or without the TRPV4 agonist GSK1016790A or 5,6‐epoxyeicosatrienoic acid (EET).^8^ The supernatant was collected after 15 minutes, and the ratio of released ATP (15‐min stimulation/30‐min basal condition) was calculated. To block TRPV4 channels, cells were pretreated with the specific TRPV4 antagonist RN‐1734 (10 μM) 24 for 30 minutes. Hypo‐osmotic solution (half saline) was used as a positive control. An aliquot (200 μL) of superfusate was then mixed with 200 μL luciferin‐luciferase reagent for luminometric ATP measurements.

### 2.9 Data analysis

Values for Ca^2+^ imaging, ATP measurements, and qRT‐PCR are presented as means ± SEM from three or more independent experiments. A Student's *t* test or nonparametric Bonferroni‐type multiple comparison was used. A chi‐square test was used for methylation rates and geographic characteristics. *R*‐squared was calculated with Excel software (Microsoft Corp., Redmond, WA, USA). Significance was accepted for *P*<.05.

## 3 Results

### 3.1 TRPV4 expression in normal human gastric epithelium and silencing in gastric cancer cells

**Figure 1:**
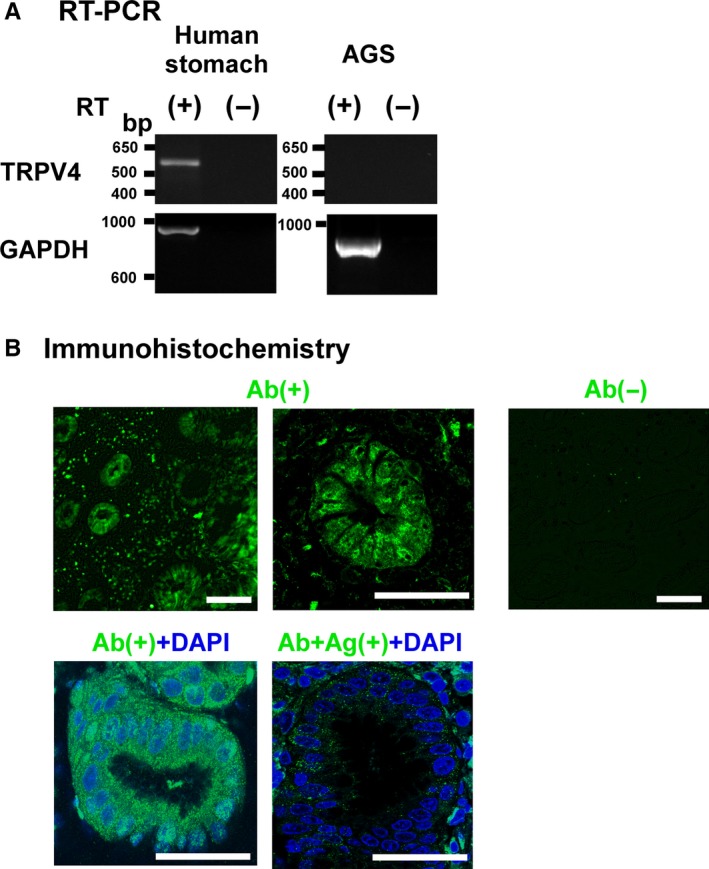
*TRPV4* mRNA and protein expression in human gastric tissues and AGS cells. A, *TRPV4* and *GAPDH* mRNA were examined with (+) and without (‐) RT reaction. The expected sizes of the amplified fragments for human *TRPV4* and *GAPDH* were 543 and 983 bp, respectively. *TRPV4* mRNA was detected in human gastric tissues but not in AGS cells. B, TRPV4‐ir was observed in human gastric epithelium. The reaction was diminished without anti‐TRPV4 antibody (Ab) or an antigenic peptide (Ag) (absorption experiment). Bars indicate 50 μm

Given that TRPV4 was shown to be expressed in the esophagus and colon epithelium, and that we recently showed that TRPV4 is expressed in mouse and rat gastric epithelia 12 and is suppressed in several cancers, we examined *TRPV4* mRNA expression in human gastric biopsy samples as well as AGS cells.^10^, ^15^, ^17^ *TRPV4* mRNA was detected in HP (‐) normal human gastric biopsy samples, but not in AGS cells (Figure 1A) or in several other gastric cancer cell lines (eg, MKN28, MKN45, data not shown). This finding is in agreement with the lack of reported TRPV4‐expressing gastric cancer cell lines in the reference database for Expression Analysis by Laboratory for Systems Biology and Medicine (http://157.82.78.238/refexa/main_search.jsp)562bp. We next examined TRPV4 protein expression in human gastric epithelium using immunohistochemistry and an absorption experiment. Our results confirmed that TRPV4 protein was indeed expressed in human gastric epithelium (Figure 1B).

### 3.2 Demethylation assay

**Figure 2:**
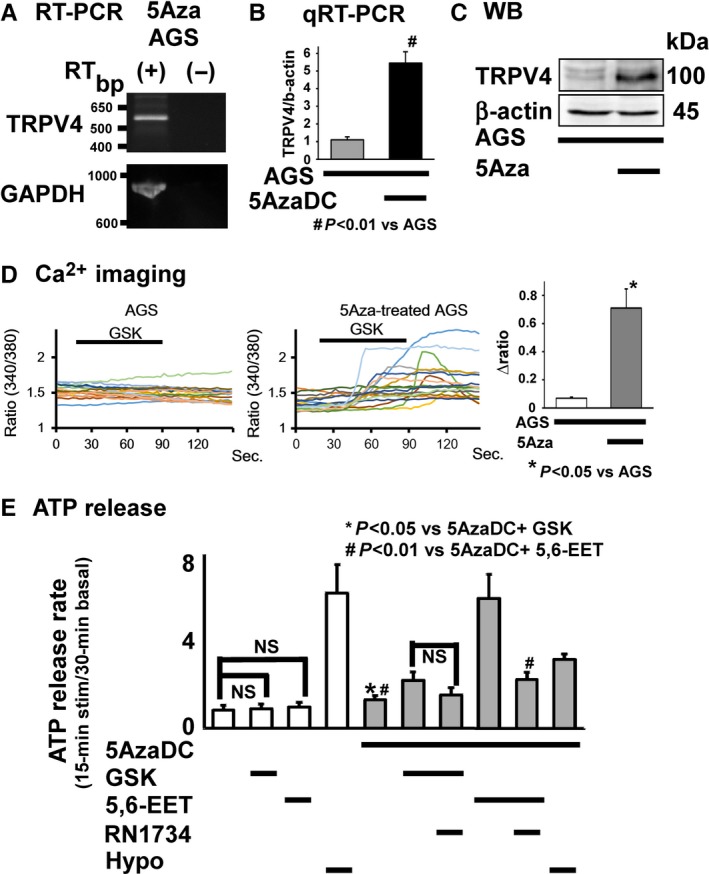
Demethylation assay. A, *TRPV4* mRNA was detected in 5Aza‐treated AGS cells. B, Quantitative RT‐PCR (qRT‐PCR) indicated significantly higher *TRPV4* mRNA expression in AGS cells treated with another demethylating agent, 5‐aza‐2′‐deoxycytidine (5‐AzaDC, 1 μM), for 1 wk compared to untreated AGS cells (# *P*<.01 vs AGS). C, Western blotting (WB) analyses revealed a band at approximately 120 kDa that is representative of the expected size for TRPV4 in AGS cells treated with 5Aza but not in untreated AGS cells. (D) Ca^2+^ imaging traces showed no [Ca^2+^]_i_ responses to the specific TRPV4 agonist GSK (100 nM) in untreated AGS cells and various [Ca^2+^]_i_ responses to the same stimuli in 5Aza‐treated AGS cells, with significantly larger responses in 5Aza‐treated AGS cells compared to untreated cells (*P<.05 vs AGS). (E) A luciferin–luciferase assay to evaluate ATP release showed that GSK1016790A (GSK, 100 nM) or 5,6‐EET (500 nM) did not enhance ATP release in AGS cells that had not been treated with 5AzaDC. AGS cells responded to a hypotonic solution as a positive control. GSK induced significantly larger ATP release from 5AzaDC‐treated AGS cells than control cells (*P<.05 vs 5AzaDC+ GSK). Pretreatment with the TRPV4 antagonist RN1734 (100 μΜ) inhibited the response (without statistical significance). 5,6‐EET also induced significantly larger ATP release from 5AzaDC‐treated AGS cells than control cells (# *P*<.01 vs 5AzaDC+ 5,6‐EET), while RN1734 pretreatment significantly inhibited this response. Values were mean + *SEM*

As recent studies suggested that *H. pylori* infection induced methylation silencing in gastric epithelium and gastric cancer cells,^4^, ^5^ we examined the effect of the demethylating agent 5‐azacytidine (5‐Aza, 10 μM) or 5‐AzaDC (1 μM) on *TRPV4* mRNA expression in AGS cells and found that *TRPV4* expression could be recovered with both demethylating agents (Figure 2A,B). TRPV4 protein expression was also recovered with 5Aza (Figure 2C). These results suggested that *TRPV4* expression might be suppressed by a DNA methylation‐dependent mechanism. We further examined whether the demethylating agents could functionally recover Ca^2+^ influx and ATP release in the presence of the specific TRPV4 agonist GSK1016790A (GSK) 9 using a fluorescent Ca^2+^ imaging system and luciferin‐luciferase assay, respectively. Ca^2+^ imaging showed significantly larger AGS cell responses to GSK (100 nM) following 5Aza treatment (Figure 2D). AGS cells showed no ATP release in response to GSK or 5,6‐EET, but had a normal response to the hypotonic solution that served as a positive control. Meanwhile, ATP release was significantly enhanced by prior 5Aza treatment, with rates that were similar to the response seen with hypotonic solution. The endogenous TRPV4 activator 5,6‐EET induced ATP release that could be inhibited by pretreatment with the TRPV4 antagonist RN1734 (10 μM) (Figure 2E), suggesting that 5,6‐EET‐induced ATP release was mediated via TRPV4. These data strongly indicated that functional TRPV4 expression could be recovered in AGS cells presumably via a demethylation‐dependent mechanism.

### 3.3 Methylation‐specific PCR in AGS cells

**Figure 3:**
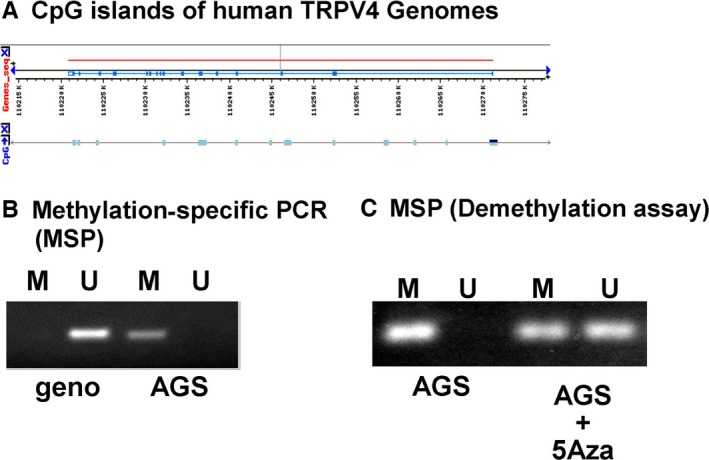
Methylation‐specific PCR (MSP). A, Black arrows, aqua boxes, and red lines indicate transcriptional orientations, CpG islands, and *TRPV4* exons, respectively. CpG islands are present in the human *TRPV4* gene, including the promoter region. B, Methylation‐specific PCR analysis of *TRPV4* gene methylation in fully unmethylated genes, GenomiPhi (geno) and AGS cells; M = methylated PCR products, U = unmethylated PCR products. *TRPV4* methylation was noted in AGS cells but not in geno. C, Incubation with the demethylating agent 5‐azacytidine (5‐Aza, 10 μM) for 1 wk partially demethylated the *TRPV4* gene

Based on recent studies suggesting that *H. pylori* infection induces methylation silencing in gastric epithelium and gastric cancer cells,^4^, ^5^ together with the presence of CpG islands in human *TRPV4* (Figure 3A) and our result showing that demethylating agents could recover functional TRPV4 expression in AGS cells (Figures 1 and 2), we hypothesized that *TRPV4* expression in human gastric epithelial cells can be suppressed by DNA methylation. We designed 21 sets of primers for methylation‐specific PCR (MSP) and selected the most relevant primers (Table S2). MSP data clearly showed that *TRPV4* is methylated in AGS cells, but not in the fully unmethylated gene, GenomiPhi (negative control) (Figure 3B). We next evaluated the demethylating effect of 5Aza (10 μM) on gene methylation status and found that TRPV4 was partially demethylated in 5Aza‐treated AGS cells (Fig. 3C). These results showed that *TRPV4* suppression could be mediated by DNA methylation in gastric epithelial cells.

### 3.4 HP‐induced direct methylation silencing of TRPV4 channel activity in normal human gastric epithelial cells

**Figure 4:**
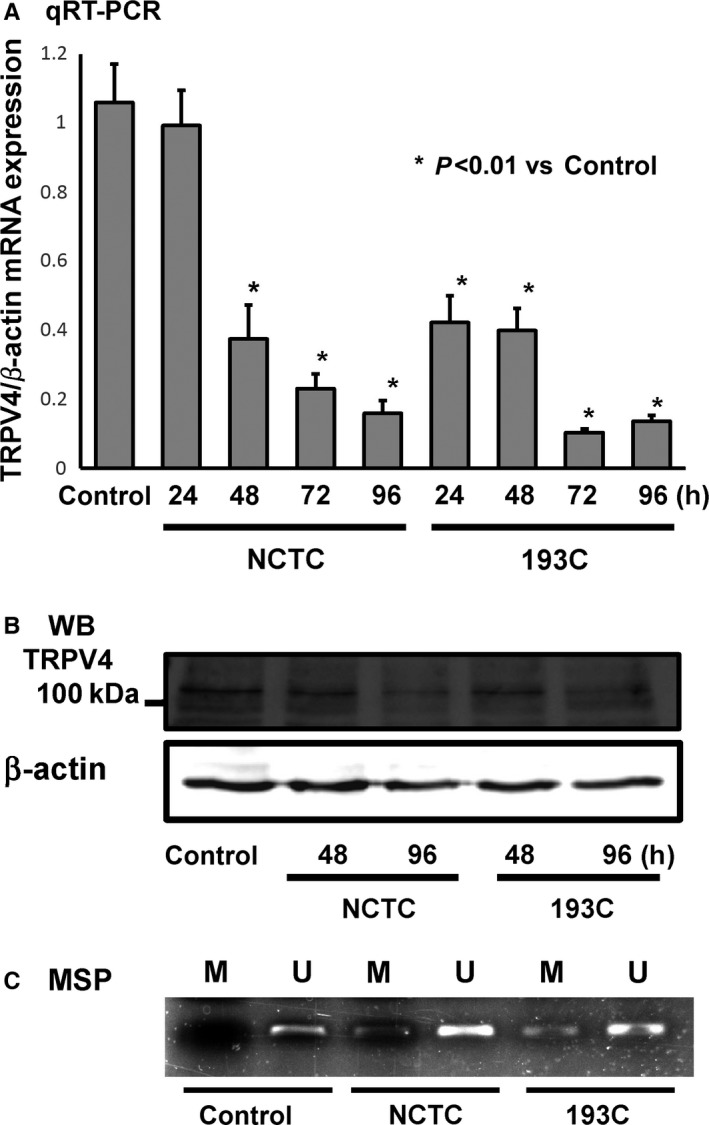
TRPV4 DNA methylation silencing in the human normal gastric epithelial cell line GES‐1 with *Helicobacter pylori* coculture. A, *TRPV4* mRNA expression levels in GES‐1 with coculture of two different types of *H. pylori* (NCTC and 193C). Relative expression levels significantly decreased in a time‐dependent manner with coculture of both *H. pylori* types (*P<.01 vs control). B, Western blotting (WB) indicated that TRPV4 protein expression also decreased with coculture of *H. pylori* in a time‐dependent manner. C, MSP indicated that methylated PCR products (M) were observed in coculture GES‐1 samples, but not in the control

Next, we evaluated the direct influence of HP coculture on TRPV4 methylation silencing in the noncancerous human gastric epithelial cell line GES‐1. Coculture of GES‐1 cells with HP (NCTC or 193C) gradually decreased *TRPV4* mRNA expression levels (Figure 4A). TRPV4 protein levels in GES‐1 cells also decreased after 96 hours of HP coculture (Figure 4B). MSP indicated that *TRPV4* in GES‐1 cells was originally fully unmethylated, but after 96 hours of HP coculture, *TRPV4* methylation was induced (Figure 4C). These data clearly showed that HP had direct consequences on *TRPV4* methylation silencing in human gastric epithelium.

### 3.5 *TRPV4* methylation silencing in human biopsy samples with *H. pylori* infection

**Figure 5:**
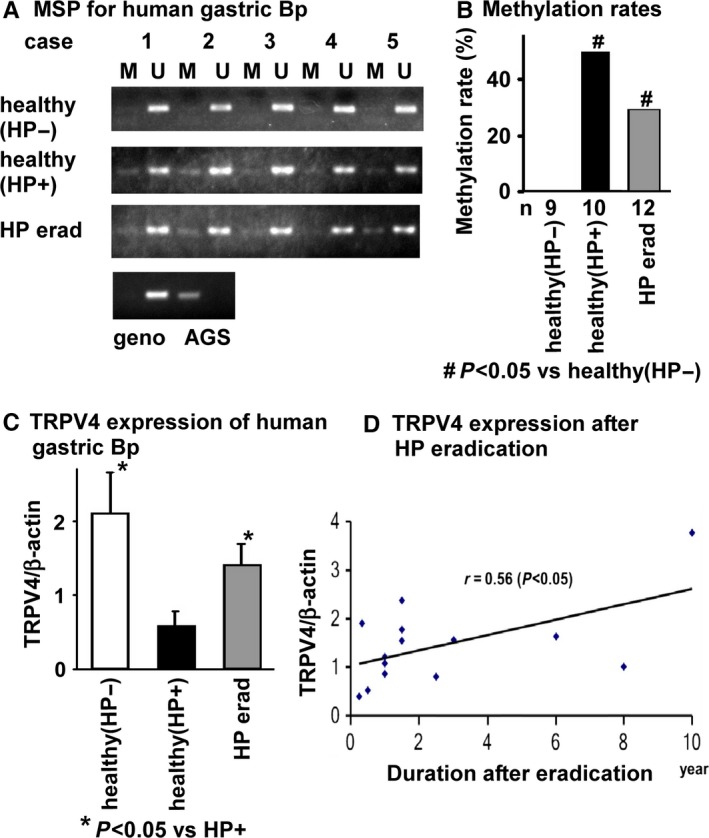
TRPV4 DNA methylation silencing in *Helicobacter pylori*‐infected human gastric stomach tissue. A, MSP for the *TRPV4* gene in *H. pylori‐*negative (HP−), *H. pylori*‐positive (HP+), or *H. pylori*‐eradicated (erad) gastric epithelium. Methylated PCR products (M) were observed in some HP+ or HP erad samples, but never in HP− samples. B, Methylation rates (%) were significantly higher in samples from HP+ healthy or HP erad individuals compared to HP− subjects (#*P*<.05 vs healthy [HP−]). C, *TRPV4* mRNA expression levels in human gastric biopsy samples. Significantly higher TRPV4 expression was detected in samples from healthy (HP−) and HP erad individuals than in healthy (HP+) subjects (*P<.05 vs HP+). D, *TRPV4* expression levels plotted relative to the duration after HP eradication (y) indicated a weak correlation between the time after HP eradication and *TRPV4* expression levels (*r*=.56, *P*<.05, n=14)

To examine the association between HP infection and *TRPV4* methylation silencing in human stomach tissue, we compared *TRPV4* methylation status and mRNA expression levels in human biopsy samples obtained from HP‐negative (−) and HP‐positive (+) healthy individuals, as well as in tissues from patients where HP infection was successfully eradicated (HP erad; patient characteristics are summarized in Table 1). Methylation‐specific PCR products (M) were observed in HP+ or HP erad samples, but never in HP‐ samples (Figure 5A). Similarly, the methylation rate (%) was significantly higher in samples obtained from HP+ individuals or HP erad patients than HP‐ individuals (Figure 5B, *P*<.05). Meanwhile, significantly lower *TRPV4* mRNA expression was noted in samples obtained from HP+ compared to HP‐ or HP erad patients (Figure 5C). Plotting *TRPV4* expression levels relative to the duration after HP eradication showed a weak proportional relationship (Figure 5D, n=15, *r*=.56, *P*<.05).

## 4 Discussion

We showed TRPV4 expression in human gastric epithelium and the noncancerous gastric epithelial cell line, GES‐1, as well as methylation‐dependent gene silencing in the human gastric cancer cell line, AGS (Figure 1, 2, 3). We also showed that *TRPV4* in GES‐1 could be directly silenced with HP coculture (Figure 4). Furthermore, TRPV4 suppression in HP‐infected individuals might be recovered by HP eradication presumably in a time‐dependent manner (Figure 5).

While there is a growing amount of evidence concerning TRPV4 channelopathies (a heterogeneous group of disorders resulting from ion channel dysfunction),^16^ and particularly for hereditary mutations in channel genes that alter channel function, to our knowledge, this is the first report that shows ion channel gene suppression via a HP‐induced DNA methylation silencing mechanism.

We previously reported that TRPV4 is expressed in mouse and rat gastric epithelium where it contributes to ATP release and gastric emptying.^12^ Current data suggested that HP‐infected individuals experience abnormal detection of gastric pressure and gastric sensory or motor dysfunction. Functional dyspepsia (FD) is characterized by continuous or frequently recurring epigastric pain or upper abdominal discomfort for which no organic cause can be determined. Although the etiology of FD remains largely unknown, a Cochrane meta‐analysis calculated that HP eradication had a small, but significant, effect (RR=0.91; 95% CI: 0.86‐0.95) in reducing dyspepsia as compared to placebo, with a need‐to‐treat (NNT) number of 15^25^ and recent higher resonance rates (NNT: 6) reported in Asian countries.^26^, ^27^ More recently, HP‐positive dyspepsia patients are diagnosed as having HP‐associated dyspepsia (HpD) if successful eradication is followed by long‐term sustained remission (6 months or longer).^3^, ^28^, ^29^ Although HP infection is not directly related to gastric emptying in human studies,^30^, ^31^, ^32^, ^33^, ^34^ several reports demonstrated that symptoms were resolved after HP eradication with normalization of gastric emptying 35, 36.This apparent contradiction could be explained with the thinking that gradual motor dysfunction can be compensated but rapid recovery is more easily detectable. We evaluated gastric biopsy samples from asymptomatic individuals, and our data limit the comparison of TRPV4 expression levels and DNA methylation rates in different individuals. In future studies, HpD patients should be targeted for prospective evaluation, especially in Asian countries.^37^

Our results suggest that TRPV4 methylation silencing could be induced not only in other inflammatory conditions in the stomach but also in other cell types. For instance, in the bladder, skin, and muscle, as well as tissues affected by skeletal and neuronal disorders, acquired *TRPV4* DNA methylation silencing might be a diagnostic and therapeutic target to treat these disorders.^16^, ^17^, ^18^

In conclusion, we showed that TRPV4 is functionally expressed in human gastric epithelium and contributes to ATP release. *TRPV4* expression is directly suppressed by DNA methylation silencing with *H. pylori* infection in gastric epithelium, suggesting that TRPV4 methylation silencing could be a novel diagnostic and therapeutic target for HpD and other functional disorders and cancers.

## Acknowledgements and Disclosures

We thank D. Kidane (The University of Texas at Austin) for his generous gift of GES‐1 cells and T. Kozawa (U. Toyama) for her technical assistance. We gratefully acknowledge the contributions of our patients and volunteers. This work was partially supported by grants to H. Mihara from the University of Toyama, Dainippon Sumitomo Corp. and JSPS KAKENHI grant number 26870214.

## Competing Interests

The authors have no competing interests.
