# Supplementary material for: Transient receptor potential vanilloid 4 (TRPV4) silencing in Helicobacter pylori‐infected human gastric epithelium
Source: Helicobacter. 2016 Sep 30;22(2):e12361. doi: 10.1111/hel.12361 (PMC5363345; doi:10.1111/hel.12361)
Supplement: Supplementary file 1 [file HEL-22-na-s001.docx]

**Table 1.**　**Study subject demographic characteristics.**

|  | **HP-** | **HP+** | **Erad** | ***p* value** |
| --- | --- | --- | --- | --- |
| **n** | **9** | **10** | **12** |  |
| **Sex (m;%)** | **77.8** | **70.0** | **75.0** | ***NS*** |
| **Age (median)** | **47-69 (56)** | **57-77 (63)** | **37-71 (65.6)** | **NS** |

Supplementary Table 1. Primer sequences for RT-PCR, qRT-PCR and MSP.

Supplementary Table 2. Primary and secondary antisera for immunochemistry or western blotting.

**Supporting Table 1. Primer sequences for RT-PCR, qRT-PCR, and MSP.**

Primer sequences for RT-PCR.

Primer name Sequence (5′→3′)

hTRPV4-F ACATTGTCAACTACCTGACGG

hTRPV4-R ACAGGTAGGAGACCACGTTG

hGAPDH-F TGAAGGTCGGAGTCAACGGATTTGT

hGAPDH-R CATGTGGGCCATGAGGTCCACCAC

Primer sequences for qRT-PCR.

Primer name Sequence (5′→3′)

hTRPV4-F TCATGATCGGCTACGCTTCA

hTRPV4-R CCTCATTGCACACCTTCATGTT

hb-actin-F TGGCACCCAGCACAATGAA

hb-actin-R CTAAGTCATAGTCCGCCTAGAAGCA

Primer sequences for MSP.

Primer name Sequence (5′→3′)

hTRPV4 M-F TTTAGTCGAGGTTTTTTCGC

hTRPV4 M-R TAATACCTTCCGTCTCCCG

hTRPV4 U-F GGTTTAGTTGAGGTTTTTTTGT

hTRPV4 U-R CCTAATACCTTCCATCTCCCA

**Supporting Table 2. Primary and secondary antisera for immunochemistry (IC) and western blotting (WB).**

Tissue antigen Host Dilution Source

TRPV4 Rabbit 1:500 (IC) or 1:1000 (WB) Abcam

β-actin Goat 1:2000 (WB) Santa Cruz Biotechnology, Inc.

Secondary antibodies used for immunochemistry or western blotting.

Antibody label Dilution Source

Goat anti-rabbit IgG-Alexa488 1:1500 Invitrogen, Inc.

Anti-rabbit Ig/HRP 1:2000 Dako

Anti-goat Ig/HRP 1:2000 Dako
